# Low tumor necrosis factor‐α levels predict symptom reduction during electroconvulsive therapy in major depressive disorder

**DOI:** 10.1002/brb3.933

**Published:** 2018-02-22

**Authors:** Annamari Sorri, Kaija Järventausta, Olli Kampman, Kai Lehtimäki, Minna Björkqvist, Kati Tuohimaa, Mari Hämäläinen, Eeva Moilanen, Esa Leinonen

**Affiliations:** ^1^ Department of Psychiatry Tampere University Hospital Tampere Finland; ^2^ Department of Psychiatry School of Medicine University of Tampere Tampere Finland; ^3^ Department of Psychiatry Seinäjoki Hospital District Seinäjoki Finland; ^4^ Department of Neurosurgery, Neurology and Rehabilitation Tampere University Hospital Tampere Finland; ^5^ The Immunopharmacology Research Group Faculty of Medicine and Life Sciences University of Tampere and Tampere University Hospital Tampere Finland

**Keywords:** cytokine, electroconvulsive therapy, major depressive disorder, seizure

## Abstract

**Objective:**

Changes in the tumor necrosis factor‐α (TNFα) have been associated with major depressive disorder (MDD). Findings concerning the effects of electroconvulsive therapy (ECT) on the TNFα level have been contradictory. The aim was to examine the immediate and long‐term changes in the TNFα level and their associations with symptom reduction in patients with MDD during ECT.

**Method:**

The study included 30 patients with MDD. Their TNFα levels were measured at baseline and 2 and 4 hr after the first, fifth and last ECT session. Depressive symptoms were assessed with the Montgomery‐Asberg Depression Rating Scale (MADRS).

**Results:**

The TNFα level decreased from baseline to the 2‐ and 4‐hr measurements. There was a correlation between the first ECT session TNFα levels and the relative symptom reduction according to the MADRS score after the ECT series. Both the first (baseline) ECT and 4‐hr TNFα levels were lower in responders than in nonresponders.

**Conclusion:**

ECT consistently induced a decrease in the TNFα level after each studied session. A low TNFα level at the first ECT appeared to predict a symptom reduction. These findings suggest that TNFα might have a role in the pathogenesis in MDD and in the mechanism of action of ECT.

## SIGNIFICANT OUTCOMES

These findings support the role of cytokines, especially TNFα, in depression.

ECT decreased TNFα levels during each ECT session.

Low TNFα levels during the first ECT session predict symptom reduction after the treatment.

## LIMITATIONS

The number of patients is relatively small.

TNFα levels of normal controls were not available.

Plasma TNFα levels may be affected by anesthesia during ECT series.

## 1 INTRODUCTION

Electroconvulsive therapy (ECT) is considered to be the most efficacious treatment for severe major depressive disorder (MDD) (UK ECT Review Group, 2003). Thus, the main indications for ECT are severe psychotic MDD and treatment resistance in patients with MDD (American Psychiatric Association, 2001). However, the mechanism of action of ECT remains obscure.

Tumor necrosis factor‐α (TNFα) is a proinflammatory cytokine that has been considered to be expressed in the brain only in response to pathological stimuli. However, TNFα as well as some other cytokines have recently been found to play an active role in neural plasticity and neurogenesis (Khairova, Machado‐Vieira, Du, & Manji, 2009; Yirmiya & Goshen, 2011). The cytokine hypothesis of depression proposes that activation of the inflammatory system, which results in increased production of proinflammatory cytokines, contributes to the pathogenesis of MDD (Maes, 1995). In animal models, cytokine activation has produced depressive symptoms, a decreased hippocampal volume, and “sickness behavior” (Dunn, Swiergiel, & de Beaurepaire, 2005; Goshen et al., 2008; Lawson, McCusker, & Kelley, 2013). Immunotherapy with interleukin‐2 or interferon‐α is reportedly associated with cognitive impairment and a depressed mood as well as fatigue, sleep disturbances, irritability, and loss of appetite (Bonaccorso et al., 2001, 2002; Capuron, Ravaud, & Dantzer, 2001; Capuron et al., 2001; Dieperink, Willenbring, & Ho, 2000). Vaccines, which act as cytokine inducers, also cause a depressed mood in healthy individuals (Harrison et al., 2009).

Proinflammatory cytokines such as TNFα are associated with the actions of mood‐relevant neurotransmitters, including the monoamines, by different pathways (Miller, Maletic, & Raison, 2009). However, the reported associations between TNFα and MDD or ECT are inconsistent due to different methodologies and variations in study populations. In addition, both plasma and serum TNFα levels have been reported. Several meta‐analyses have revealed markedly higher concentrations of TNFα in depressed individuals than in healthy controls (Dowlati et al., 2010; Goldsmith, Rapaport, & Miller, 2016; Huang & Lee, 2007; Liu, Ho, & Mak, 2012). However, studies with no association with changes in the TNFα level and symptoms of MDD have been published (Haapakoski, Mathieu, Ebmeier, Alenius, & Kivimäki, 2015; Karlovic, Serretti, Vrkic, Martinac, & Marcinko, 2012; Yoon, Kim, Lee, Kwon, & Kim, 2012). This association has been questioned in a recent meta‐analysis (Anisman, Ravindran, Griffiths, & Merali, 1999). It has also been hypothesized that the pathophysiological mechanisms of various subtypes of MDD might differ from one another with respect to dysregulation of the immune‐inflammatory system (Lamers et al., 2013). One report suggested that atypical depression is associated with an elevated TNFα level (Janelidze, Mattei, Westrin, Träskman‐Bendz, & Brundin, 2011). Elevated TNFα levels have also been associated with suicide attempts and dissociative symptoms (Abbasi, Hosseini, Modabbernia, Ashrafi, & Akhondzadeh, 2012; Bizik et al., 2014). The nonsteroidal anti‐inflammatory drug celecoxib reportedly has a beneficial effect in patients with MDD as an adjuvant medication (Akhondzadeh et al., 2009; Kargar et al., 2014; Müller et al., 2006). Additionally, celecoxib reduced the TNFα level after an ECT series in patients with bipolar disorder (Dantzer, O'Connor, Lawson, & Kelley, 2011).

It has been presumed that TNFα, through its induction of indoleamine 2,3‐dioxygenase (IDO), activates the kynurenine pathway, which is considered to be associated with inflammation and depression. IDO catabolizes tryptophan into neuroactive kynurenine metabolites such as quinolic acid and 3‐hydroxy kynurenine. These tryptophan metabolites are neurotoxic because they are able to generate oxidative radicals and act as N‐methyl‐N‐aspartate receptor agonists (Haroon, Raison, & Miller, 2012; Maes, Mihaylova, Kubera, & Ringel, 2012; Schwarcz, Bruno, Muchowski, & Wu, 2012). Another metabolite of kynurenine, kynurenic acid, is considered to have neuroprotective properties (Haroon et al., 2012; Myint & Kim, 2003). It has been suggested that in patients with depression, activation of IDO could turn the kynurenine pathway toward the generation of neurotoxic metabolites (Guloksuz et al., 2015). ECT was recently reported to increase the levels of kynurenic acid, referring to the fact that ECT can alter the balance of the kynurenine pathway toward a neuroprotective course (Schwieler et al., 2016). Furthermore, ECT can reportedly decrease quinolic acid levels in patients with treatment‐resistant depression (Hestad, Tønseth, Støen, Ueland, & Aukrust, 2003). Altogether, these findings imply that the mechanism of action of ECT is partly associated with the kynurenine pathway, which is induced by TNFα.

Some studies have addressed the association between TNFα and ECT (First, Spitzer, Gibbon, & Williams, 1996; Fluitman et al., 2011; Hestad et al., 2003; Rotter et al., 2013; Rush et al., 2016; Zincir, Ozturk, Bilgen, Izci, & Yukselir, 2016). In 2003, Hestad et al. (Fluitman et al., 2011) reported a decrease in the TNFα level in patients with MDD along with clinical improvement during and after an ECT series, whereas another study revealed a rapid increase in the TNFα level after a single ECT session but no long‐term change during the ECT series (Rotter et al., 2013). Other studies have found no change in the TNFα level during or after the ECT series (First et al., 1996; Hestad et al., 2003; Zincir et al., 2016). A recent study revealed a decrease in the TNFα level during and after an ECT series, although the baseline TNFα level before ECT was lower in patients than in controls (Rush et al., 2016).

### 1.1 Aims of the study

The causality of the TNFα level and ECT as well as the association between changes in the TNFα level and symptoms during ECT are contradictory. The aim of this study was to examine the changes in the plasma level of TNFα and their possible associations with symptom reduction in patients with MDD at three different sessions during an ECT series. These sessions were included to study the immediate and long‐term effects of ECT on the TNFα level during an ECT series. TNFα was measured from plasma (instead of serum) in order to avoid artifact production of TNF alpha during the sampling procedure/clotting process (de Jager, Bourcier, Rijkers, Prakken, & Seyfert‐Margolis, 2009).

## 2 MATERIAL AND METHODS

### 2.1 Clinical characteristics

Forty‐nine patients with severe or psychotic MDD who were consecutively admitted for ECT to the Department of Psychiatry, Tampere University Hospital were asked to participate in this study. Thirteen of these 49 patients declined, and six patients withdrew from the study. Therefore, the study group comprised 30 patients (12 female, 18 male). On admission, the Structured Clinical Interview for DSM‐IV Disorders (American Psychiatric Association, 1994) was conducted to confirm the diagnosis in each patient. All patients fulfilled the DSM‐IV diagnostic criteria for MDD (Montgomery & Åsberg, 1979), and 14 of them had psychotic symptoms. Nine patients suffered from a first episode of MDD, and the MDD was recurrent in 21 patients.

Patients with bipolar depression and major psychiatric disorders other than MDD were excluded. Patients with progressive organic brain disorders, epilepsy, inflammatory or autoimmune diseases, and alcohol or other substance abuse were also excluded from the study. Patients who had undergone ECT within 3 months prior to entry into the study were not included.

The mean age of the patients was 57.1 years (standard deviation [*SD*], 17.7; range, 25–85 years). The patients underwent constant psychotropic drug treatment throughout the entire ECT period (Table 1). Twenty‐eight patients in the study were taking a combination of at least two psychotropic medications, and only two were undergoing monotherapy. Benzodiazepines were discontinued 10 hours before ECT to avoid any effects on the seizure threshold.

**Table 1:** Psychotropic medications of patients with MDD during the ECT series

	All patients (*n* = 30)	Female patients (*n* = 12)	Male patients (*n* = 18)
Age			
Mean ± *SD*	57.1 ± 17.7	71.1 ± 12.2	45.2 ± 17.0
Range	25–85	45–85	25–79
Total number of ECTs			
Mean ± *SD*	10.4 ± 3.6	10.8 ± 4.3	8.2 ± 3,6
Range	5–17	5–17	5–13
Psychotic symptoms	14	7	7
First episode of MDD	9	4	5
Recurrent MDD	21	13	8
Antidepressants			
SSRI	9	3	6
SNRI	12	5	7
Mirtazapine	12	6	6
Bupropion	4	1	3
Antipsychotics			
Second generation antipsychotics	28	11	17
Conventional neurolepts	1	0	1
Anxiolytics			
Benzodiazepines	21	8	13
Pregabalin	2	2	0
Buspirone	1	0	1

SSRI, Selective serotonin reuptake inhibitor; SNRI, Serotonin and norepinephrine reuptake inhibitor.

The severity of depression was quantified by the Montgomery‐Asberg Depression Rating Scale (MADRS) (Montgomery & Åsberg, 1979) in addition to the TNFα level before the first and after the fifth and last ECT session.

All patients provided written informed consent. This study design was reviewed and approved by the Tampere University Hospital Ethics Committee.

### 2.2 Electroconvulsive therapy

Before the first ECT session, a medical history was obtained and a physical examination with routine blood examination and electrocardiogram was performed. ECT was administered three times a week with a constant‐current, brief‐pulse device (MECTA spECTrum 5000Q; MECTA Corp., Lake Oswego, OR, USA). The seizure threshold was titrated during the first treatment, and subsequent treatments were administered at 1.5 times the seizure threshold for bilateral ECT. Seizures of >25 s in duration on electroencephalography were deemed adequate. If the duration was shorter, a restimulation was performed during the same treatment session with a 50% increase in the stimulus intensity.

### 2.3 Anesthesia

Anesthesia was induced with methohexital (initial dose of 1 mg/kg), and muscle relaxation was achieved with succinylcholine (initial dose of 0.5 mg/kg). The patients were ventilated with 100% oxygen until resumption of spontaneous respiration. Physiological monitoring during the treatment included pulse oximetry, blood pressure, electrocardiography, one‐channel electroencephalography and electromyography. All patients were treated with standard bilateral (bifrontotemporal) ECT. The number of treatments ranged from 5 to 17 (10.4 ± 3.6 [mean ± *SD*]). ECT treatment was discontinued on the basis of clinical judgment if the patient was either in remission (MADRS score of ≤10), or no further improvement was recorded during the last two ECT sessions. In five patients, the fifth ECT session was the last (not included in the last ECT session data).

### 2.4 Blood sampling

The blood sampling was strictly standardized. Blood was drawn before ECT (baseline) and 2 and 4 hr after ECT at the first, fifth, and last sessions, and EDTA‐treated plasma was separated and stored at −80°C until analyzed. The concentration of TNFα was determined by enzyme‐linked immunosorbent assay with commercial reagents (Quantikine HS; R&D Systems Europe, Ltd., Abingdon, UK). The detection limit and interassay coefficient of variation were 0.25 pg/ml and 3.3%, respectively.

### 2.5 Statistical methods

A paired‐sample *t*‐test was used to analyze changes between the TNFα level at different measurement points: baseline vs. 2 hr, baseline vs. 4 hr, and 2 hr vs. 4 hr. The relationships between the TNFα level and symptom reduction according to the MADRS as well as other clinical variables were examined with Spearman's rho due to the skewed distribution in the MADRS response rate variable. Comparisons between responders’ and nonresponders’ TNFα levels were performed using the Mann–Whitney *U*‐test because of the non‐normal distribution of the TNFα levels. A Bonferroni correction was used in the correlation calculation by setting the limit of statistical significance to 0.05/9, that is, by dividing by the number of tests. In all other tests, the level of significance was set at *p *<* *.05. Spearman's correlation coefficients were used in the exploratory analyses for selecting statistically significant or trend level (*p *<* *.1) correlating explanatory variables for the regression model to predict dichotomized response, in addition to the included confounding variables age and gender. A logistic regression model was used to predict a dichotomized response. In the model, TNF alpha baseline value, number of ECT treatments, gender, and age were used as explanatory variables. Odds ratios (OR) and 95% confidence intervals (CI) were calculated for covariates. Statistical analyses were performed with the Statistical Package for the Social Sciences (SPSS) version 22.0.

## 3 RESULTS

**Figure 1:**
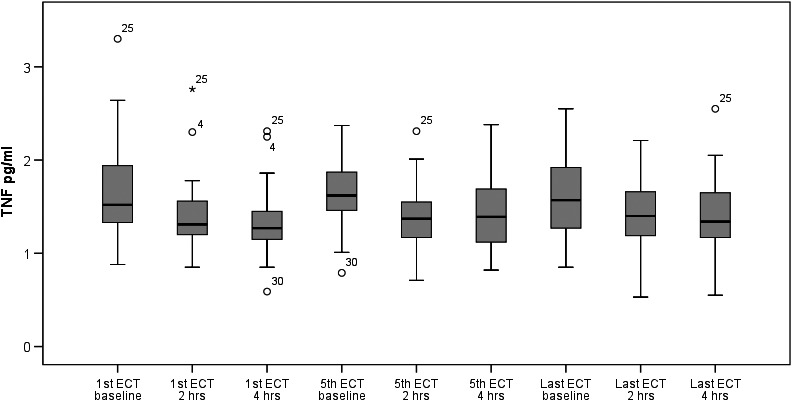
Plasma TNFα levels at first, fifth, and last ECT sessions in patients with MDD

The plasma levels of TNFα during ECT series are presented in Table 2. These levels consistently decreased from baseline (before ECT) to 2 hr during the first (*p *<* *.001), fifth (*p *<* *.001), and last (*p *=* *.007) ECT sessions. The plasma TNFα level also decreased from baseline to 4 hr during every ECT session (*p *<* *.001, *p *<* *.001, and *p *=* *.001, respectively). No significant changes were found in the TNFα level between the 2‐ and 4‐hr measurements. When comparing the baseline TNFα level among the first, fifth, and last ECT sessions, no significant changes were found in the whole study group (Figure 1).

**Table 2:** Means, standard deviations, and ranges of plasma TNFα levels during ECT series (pg/ml)

ECT	Measurement	*N*	Mean	Standard deviation	Range
First	Baseline	30	1.65	0.56	2.42
	2 hr	30	1.42	0.47	1.91
	4 hr	30	1.34	0.43	1.88
Fifth	Baseline	30	1.64	0.45	2.23
	2 hr	30	1.41	0.39	1.76
	4 hr	30	1.48	0.46	1.82
Last	Baseline	25	1.59	0.43	1.70
	2 hr	25	1.42	0.39	1.68
	4 hr	25	1.39	0.43	2.00

**Figure 2:**
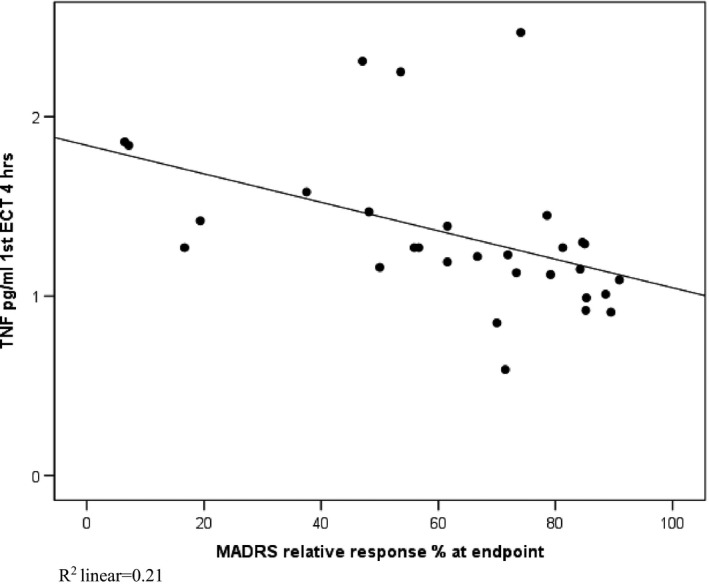
First ECT 4‐hr TNFα levels and relative symptom reduction in MADRS. *R* ^*2*^ linear = .21

Prior to the first ECT session, the mean MADRS score was 31.6 ± 7.2 (mean ± *SD*), and that after the ECT series was 11.3 ± 7.5. At the end of the study, 22 of 30 patients showed at least a 50% decrease in MADRS score, of those 20 were in remission (MADRS score of ≤10). There was a significant correlation between the baseline TNFα level at the first ECT session (prior to ECT series), the 2‐ and 4‐hr TNFα levels, and the relative (%) symptom reduction after the ECT series as reflected by the MADRS score (ρ = −.588, *p *=* *.001; ρ = −.574, *p *=* *.001; ρ = −.600, *p *<* *.001, respectively) (Table 3). Low TNFα concentrations and symptom reduction according to the MADRS score were interrelated (Figure 2). These correlations were inconsistent in subsequent ECT sessions (fifth and last). The TNFα level at both the first ECT session (baseline) and 4 hr were lower in responders (50% symptom reduction) than in nonresponders (*p *=* *.035 and *p *=* *.006, respectively). The dichotomized response had a statistically significant or trend level (*p *<* *.1) correlation with smaller number of ECT treatments (ρ = −.32, *p *=* *.084) and with lower baseline TNFα levels (ρ = −.39, *p *=* *.032). The best fitting logistic regression model for predicting the dichotomized response (*p *=* *.03) included TNF alpha baseline value (OR = 0.20, 95% CI 0.04–1.15, *p *=* *.07) and number of ECT treatments (OR = 0.76, 95% CI 0.57–1.02, *p *=* *.07) as explanatory variables. This model explained 31.0% of the outcome variance (Nagelkerke R square); the sensitivity for response was 90.9% and specificity 50%. Gender and age had nonsignificant correlations with the dichotomized response. Neither gender nor age had any predictive effect on the response, and omitting these variables from the model did not change the predictive values.

**Table 3:** Correlations between plasma TNFα levels and relative (%) symptom reduction in patients with MDD during ECT

ECT	Measurement	rho	*p*
First	Baseline	−.558	.001^a^
	2 hr	−.574	.001^a^
	4 hr	−.600	.001^a^
Fifth	Baseline	−.283	.130
	2 hr	−.191	.312
	4 hr	−.284	.128
Last	Baseline	−.503	.10
	2 hr	−.390	.054
	4 hr	−.455	.022

a Significant after Bonferroni's correction.

No correlations were found between clinical parameters (e.g., duration of convulsions, heart rate variability, or changes in blood pressure during ECT) and the TNFα level or clinical outcome.

## 4 DISCUSSION

The most interesting finding in the present study was that a low TNFα level at the first ECT (0, 2, and 4 hr) appeared to predict a reduction in symptoms. No similar correlations were found during subsequent sessions. No findings suggesting associations between a low TNFα level and symptom reduction during ECT have been reported in previous studies (First et al., 1996; Fluitman et al., 2011; Hestad et al., 2003; Rotter et al., 2013; Rush et al., 2016; Zincir et al., 2016). If replicated in the future studies, this finding supports the role of TNFα in the pathogenesis of MDD and in the mechanism of action of ECT; it may also guide the selection of treatment modalities for patients with severe MDD. There has been a wide variation in TNFα levels in different studies. However, some of these have reported similar ranges of plasma TNFα levels in depressed patients compared to those in the present study (Dome et al., 2009; O′Donovan et al., 2013).

The decrease in the plasma TNFα level after each session was a consistent finding in the present study. This was observed in every studied session between the baseline measurement and both the 2‐ and 4‐hr measurements. The effect of ECT on the TNFα level appears to be transitory because the TNFα level returned to the basic level before next ECT session. This finding differs from some previous reports in which the TNFα level either increased, did not change, or decreased by ECT (First et al., 1996; Fluitman et al., 2011; Hestad et al., 2003; Rotter et al., 2013; Rush et al., 2016; Zincir et al., 2016). The design of the study by Hestad et al. (Fluitman et al., 2011) somewhat resembles the design of the present study. In their study, the TNFα level was measured during the ECT series at baseline and 1 hr after the first, fourth, and last ECT session. They reported a decrease in the baseline TNFα level during the ECT series and 24 hr and 1 week after the last ECT session (Fluitman et al., 2011). Additionally, a decrease in the TNFα level was observed in a study in which measurements were taken 1 day before the ECT series, at the time of the clinical response to ECT, and 1 day after the series (Rush et al., 2016). In contrast, Fluitman et al. (Rotter et al., 2013) reported an immediate increase in the TNFα level after every single session of ECT but found no constant change in the baseline TNFα level during the ECT series. The TNFα levels in these two latter studies were serum (not plasma) levels, making comparison with the present results difficult. Moreover, three studies found no change in the serum/plasma/plasma (respectively) TNFα level during and after the ECT series (First et al., 1996; Hestad et al., 2003; Zincir et al., 2016). However, in these studies, the timing of the blood samplings and procedures were somewhat different, hampering comparison with the present results (First et al., 1996; Hestad et al., 2003; Zincir et al., 2016). Unfortunately, the post‐ECT TNFα levels (e.g., 1 week after the series) were not available in the present study. However, if we would have observed a persistent decrease in the TNFα level after the ECT series, we would have presumed that this decrease was already apparent in the baseline measurements in different sessions during the ECT series.

Decreases in the TNFα level by anesthetics and muscle relaxant drugs (methohexital and succinylcholine) administered during ECT have been reported in patients with MDD (Stelzhammer et al., 2011). Whether this decrease found in the present study in the TNFα level is related to the anesthetics used or the anti‐inflammatory mechanisms of ECT are inconclusive and require further research.

Immune dysregulation and activation of the inflammatory response system are involved with MDD (Miller et al., 2009). Several studies have reported elevated TNFα levels in the blood and cerebrospinal fluid in patients with MDD (Dowlati et al., 2010; Goldsmith et al., 2016; Huang & Lee, 2007; Liu et al., 2012; Martinez, Garakani, Yehuda, & Gorman, 2012; Stelzhammer et al., 2011), but this finding was questioned in a recent meta‐analysis (Anisman et al., 1999). However, an elevated TNF‐α level has been somewhat associated with severe forms of depression (Anisman et al., 1999). Another meta‐analysis of inflammatory biomarkers in patients with MDD undergoing antidepressant treatment reported no decrease in the elevated baseline TNFα level. In that report, however, there was a decrease in the TNFα level in responders compared with nonresponders (Köhler et al., 2017).

The main limitation of the present study is the relatively small sample size. Additionally, the lack of healthy controls hampered the interpretation of the results. Circulating TNFα levels are affected by various metabolic and pathological conditions in addition to MDD (McArdle, Finucane, Connaughton, McMorrow, & Roche, 2013; Sinha et al., 2015). Moreover, plasma TNFα levels may be affected by anesthesia during ECT series (Stelzhammer et al., 2011). Even though the dichotomized response correlated significantly with lower baseline TNFα levels, these levels predicted the dichotomized response only at trend level in the logistic regression model. The association should therefore be confirmed in a larger sample. The baseline (before any treatment) TNFα levels used here, however, reflect only the influence of the disorder (MDD), not the effect of ECT. The strengths of this study include the standardized timing of repeated blood sampling during the individual ECT sessions and throughout the ECT series, which improves the reliability of the results. Thus, the effects of both the disorder (MDD) and the treatment (ECT) on TNFα levels have been considered.

In conclusion, low TNFα levels before and after the first ECT session predict symptom reduction after the ECT series. Every individual ECT session seemed to decrease the plasma TNFα level at 2 and 4 hr compared with baseline. This effect, however, was transitory because the baseline level did not change between the sessions. The predictive value of the first ECT session TNFα levels associated with final symptom reduction would be clinically interesting, if replicated in the further studies.

## CONFLICTS OF INTEREST

None declared.
